# Supplementary material for: Flame retardant high-power Li-S flexible batteries enabled by bio-macromolecular binder integrating conformal fractions
Source: Nat Commun. 2022 Jan 10;13:145. doi: 10.1038/s41467-021-27777-5 (PMC8748741; doi:10.1038/s41467-021-27777-5)
Supplement: Supplementary file 1 — Supplementary Information [file 41467_2021_27777_MOESM1_ESM.pdf]

Supplementary Information

Flame retardant high-power Li-S flexible batteries enabled by bio-macromolecular binder integrating conformal fractions

*Chenrayan Senthil[1], Sun-Sik Kim[1], Hyun Young Jung[1,2,]**

[1] Department of Energy Engineering, Gyeongsang National University, Gyeongnam, Jinju-si, Gyeongnam 52725, South Korea

[2] Future Convergence Technology Research Institute, Gyeongsang National University, Jinju-si, Gyeongnam 52725, South Korea

*Corresponding author:

Correspondence should be addressed to HYJ. E-mail: hyjung@gnu.ac.kr

Preparation of catholyte Li_2S_4

The catholyte solution containing Li_2S_4 in the liquid phase was prepared by adding a molar ratio of Li_2S and S, 1:3 into dimethoxymethane (DME) and 1,3-dioxolane (DOL) solution of 1:1 (v/v). Later 1.0 M lithium bis(trifluoromethane sulfonyl)imide (LiTFSI) salt was added and magnetically stirred for 12 h at 60 °C to ensure proper catholyte concentration. The static adsorption of polysulfides was studied using the concentration of 4 mM Li_2S_4 catholyte.

Shuttle factor calculation

Furthermore, shuttle factor (*f*) allows gaining insights on the degree of polysulfides shuttle during the cycles, which is expressed as[1],

$$\frac{k_s \, q_H \, [S_{\text{total}}]}{I} = f \qquad (1)$$

where k_s, q_H, $[S_{total}]$, and I denote heterogenous reaction shuttle constant, the theoretical specific capacity of the high plateau during discharge/charge process, full conversion of sulfur, and discharge/charge current, respectively. Typically, the accumulated polysulfides during discharge prolong the charging process and reduce the subsequent discharging capacity at a high plateau. Thus, the Coulombic efficiency (C_{eff}) of the discharge and charge process is accounted to reveal the shuttle factor represented as[2],

$$C_{\text{eff}} = \frac{Q_{\text{DH}} + Q_{\text{DL}}}{Q_{\text{CH}} + Q_{\text{CL}}} \qquad (2)$$

where Q_{DH}, Q_{DL}, Q_{CH}, and Q_{CL} denote the discharge and charge capacities at high and low voltage plateaus, respectively. The shuttle factor (*f*) calculated for the S/TG electrodes at the end of the 1000th cycle at 4 C was 0.07 in C_{eff} of 98.1 %, and relatively C_{eff} of 93.8 % and 92.1% for S/PVDF and S/PEO electrodes (Supplementary Fig. 5**)** led to *f* values of 0.13 and 0.19, respectively. Here, *f* approaching zero indicates a lower shuttle effect; in such case, the lower

f=0.07 clearly manifests the suppression of polysulfide shuttle, which eventually leads to a stable discharge/charge capacities of S/TG electrodes than the S/PVDF and S/PEO. Furthermore, the inability of S/PVDF or S/PEO electrodes to bind polysulfides strongly promotes the "shuttle effect," which eventually corrodes the Li anode through an irreversible reaction at the expense of capacity fade with cycles.

Li-ion diffusion coefficient

The Li-ion diffusion (D_{Li^+}) coefficient was calculated using the Randles-Sevcik equation[3,4] applied to the voltammograms obtained at various scan rates of main article Figs. 4a-c.

$$i_p=\left(2.69\times10^5\right) n^{3/2}AD^{1/2}Cv^{1/2} \qquad (3)$$

where n, A, D, C, v, and i_p represent the number of electrons, electrode area (cm^2), diffusion coefficient ($cm^2\ s^{-1}$), the concentration of Li-ion in the electrolyte (mol mL^{-1}), scan rate ($V\ s^{-1}$), and peak current (A), respectively.

Self-discharge test

Self-discharge tests were performed to ascertain the polysulfide shuttling in Li-S cells at a static condition. The Li-S cells comprising various sulfur cathodes were assembled as stated in the electrochemical characterization section. To evaluate the self-discharge property, the cells were galvanostatically cycled at the low and high current rates of 0.2 C and 1 C. The self-discharge properties of the various Li-S cells were measured after every 3 discharge-charge cycles followed by a sequential resting time of 6 h – 12 h – 24 h (1 day) – 48 h (2 days) – 72 h (3 days) and 96 h (4 days).

Wettability test

Electrode wettability test was performed using electrolyte comprised of 1 M lithium bis(trifluoromethane sulfonyl)imide (LiTFSI) salt dissolved in a eutectic anhydrous mixture

(1:1, v/v) of 1,3 dioxolane (DOL) and 1,2-dimethoxymethane (DME) with 2% lithium nitrate ($LiNO_3$). Pre-weighed calendared S/PVDF, S/PEO, and S/TG electrodes were steeped separately in an electrolytic solution for about 24 h, and the respective mass change was measured to determine the electrolyte uptake of respective electrodes.

Electrolyte/Sulfur (E/S) ratio test

Cathodes of high sulfur loading were considered for the E/S test, where the mass of S/PVDF, S/PEO, and S/TG electrodes were 2.5, 2.2, and 2.7 mg cm^{-2}, respectively. The Li-S cells comprising the above electrodes were assembled with a specified amount of E/S ratio of 6, 9, 12, and 15 μL mg^{-1} and were cycled at a current rate of 1 C.

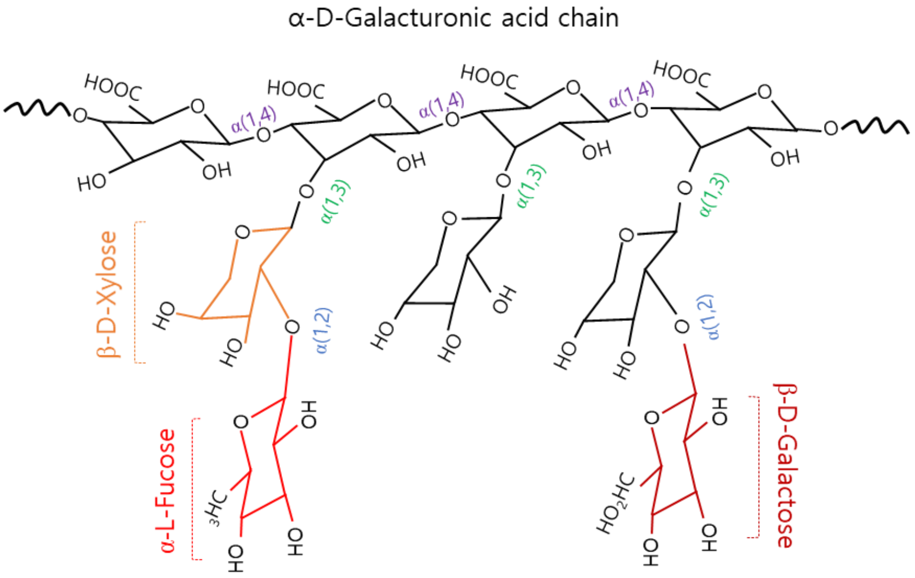

	Species	Polysaccharidic Units						
		Arabinose 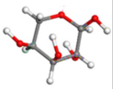	Xylose 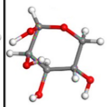	Glucose 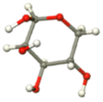	Fucose 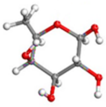	Galactose 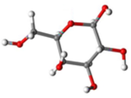	Galacturonic acid 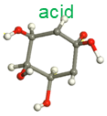	Rhamnose 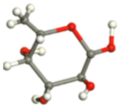
Tragacanth (Astragalus . *sp*)	*A. parrowianus*	39%	10%	10%	7%	8%	21%	-
	A. gossypinus	1%	32%	1%	23%	1%	37%	1%
	A. rabensis	51%	11%	13%	-	7%	9%	-
	A. fluccosus	23%	24%	-	8%	7%	34%	-
	A. microcephalus	35%	19%	10%	9%	2%	21%	1%
	A. compactus	7%	21%	2%	35%	2%	30%	1%

Supplementary Figure 1. Macromolecular structure proposed for tragacanth gum (TG) and the composition of various saccharadic units specific to species of Astragalus[5].

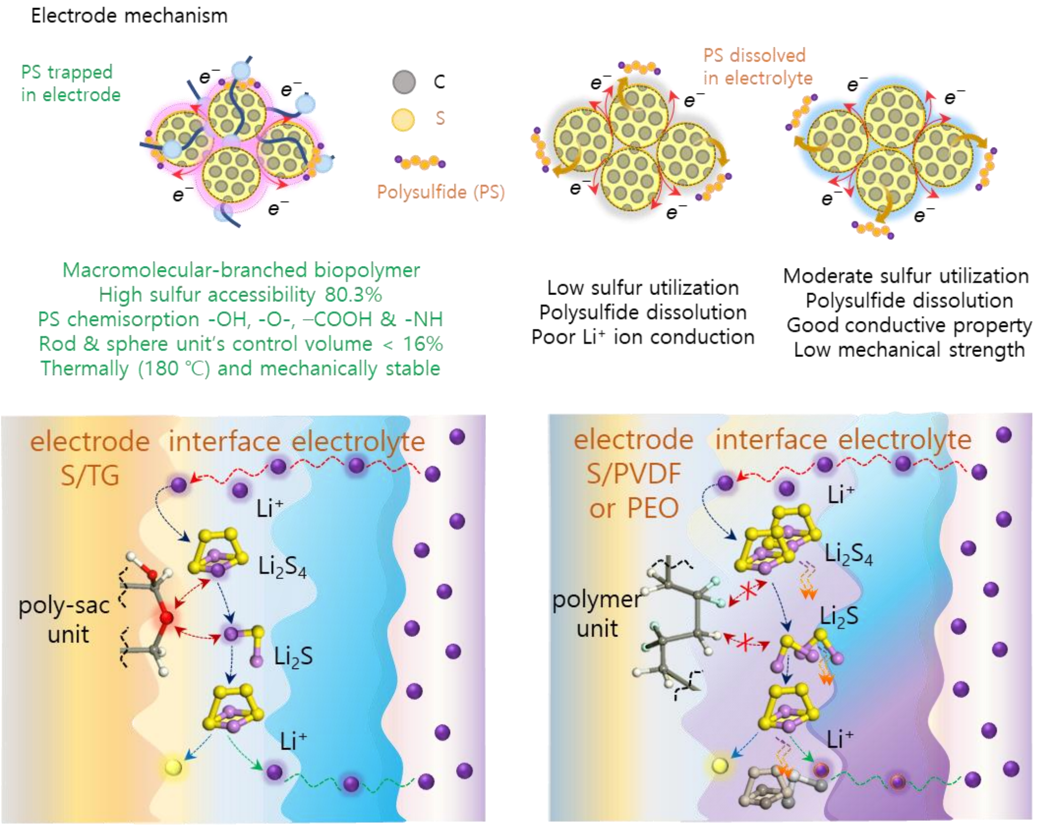

Supplementary Figure 2. Illustration depicting the electrochemical reaction mechanism that occurs in conventional S/PVDF and S/PEO electrodes and the one proposed for S/TG electrode in Li-S cells. Scheme for S/PVDFrepresents the reaction of Li with sulfur to produce Li_2S_x (x=1-8) during discharge and the reversible process in S/PVDF and S/PEO electrodes, where Li_2S_x encounters dissolution and precipitation in electrolyte. S/TG chemisorbs Li_2S_x through abundant polar functional groups, while preventing the loss of sulfur as Li_2S_x and maintaining the electrolyte concentration.

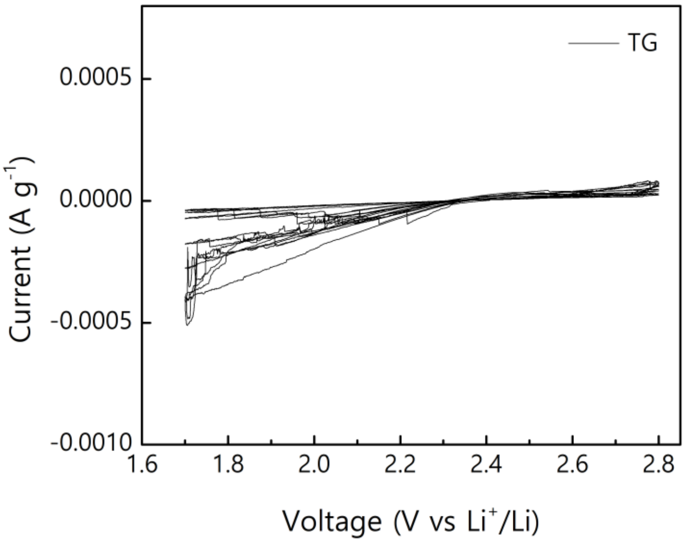

Supplementary Figure 3. Cyclic voltammogram of pure TG binder performed at 1 mV s^{-1} at a potential window 1.7 V to 2.8 V.

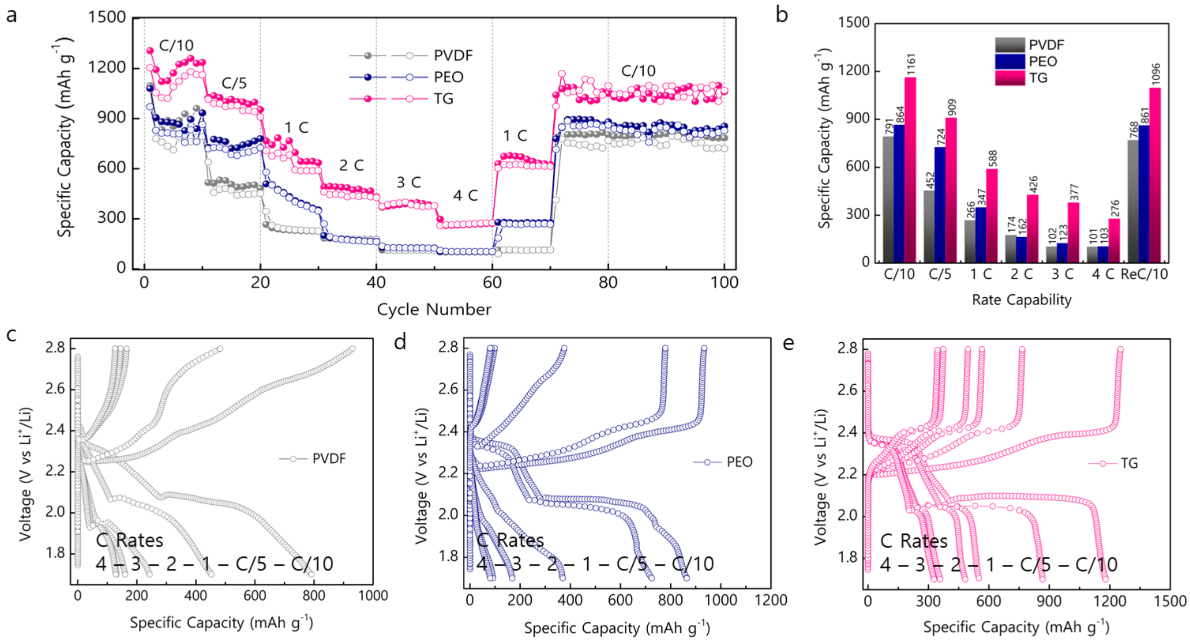

Supplementary Figure 4. Rate performance studies of S/PVDF, S/PEO, and S/TG electrodes at various current rates. **a** Cycle stability at different rates. **b** Bar graph depicting reversible capacity at the end of 10th cycle at current rates. **c-e** profile shown for S/PVDF, S/PEO, and S/TG electrodes at respective rates.

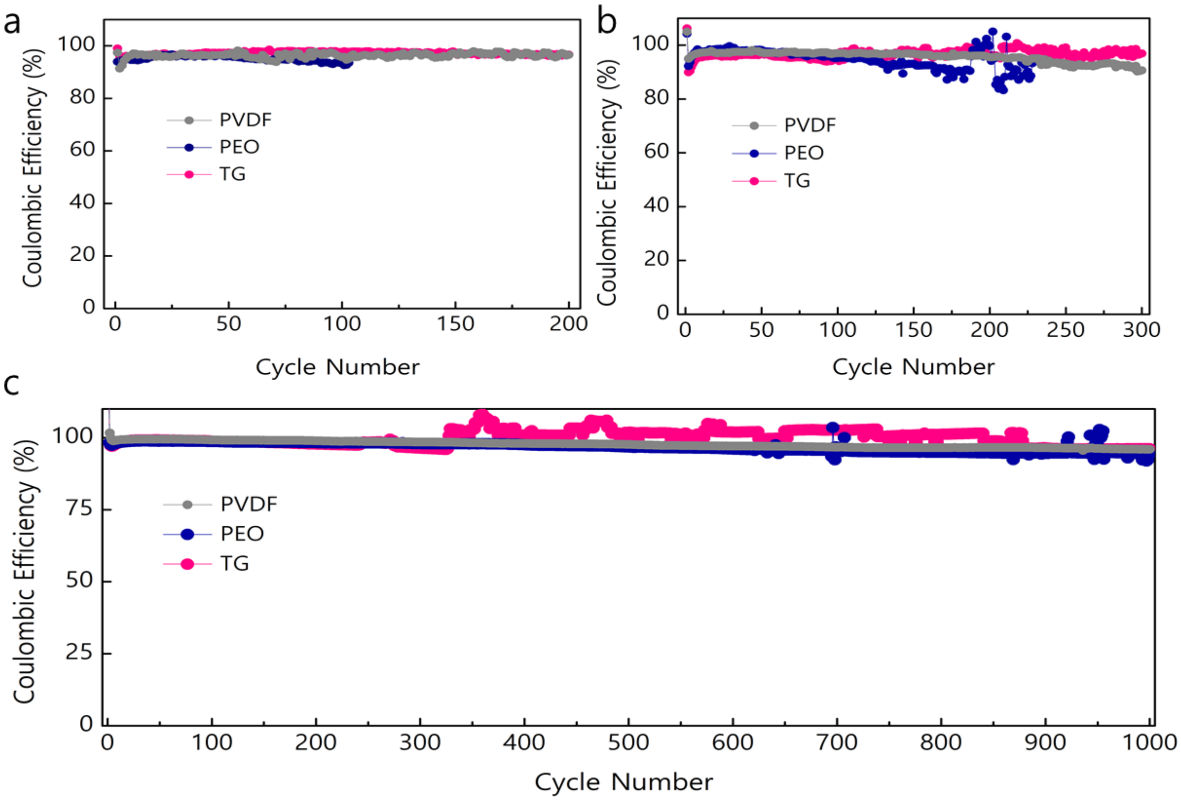

Supplementary Figure 5. Coulombic efficiency (CE) of the S/PVDF, S/PEO, and S/TG electrodes calculated from the galvanostatic discharge-charge cycling at various current rates. **a** C/5. **b** 1 C. and **c** 4 C.

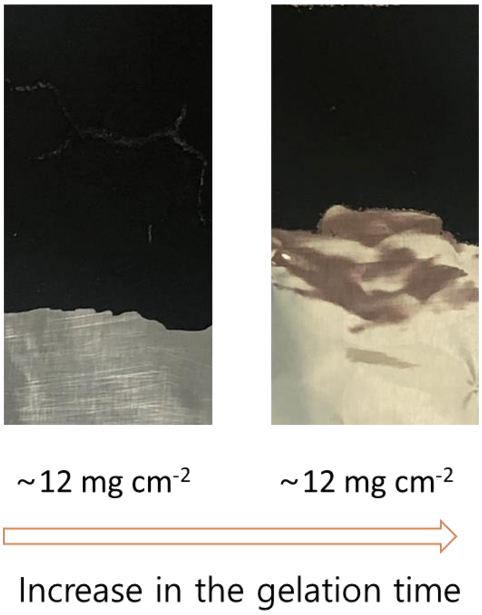

Supplementary Figure 6. Digital photographs of S/TG electrodes with highly loaded sulfur active material.

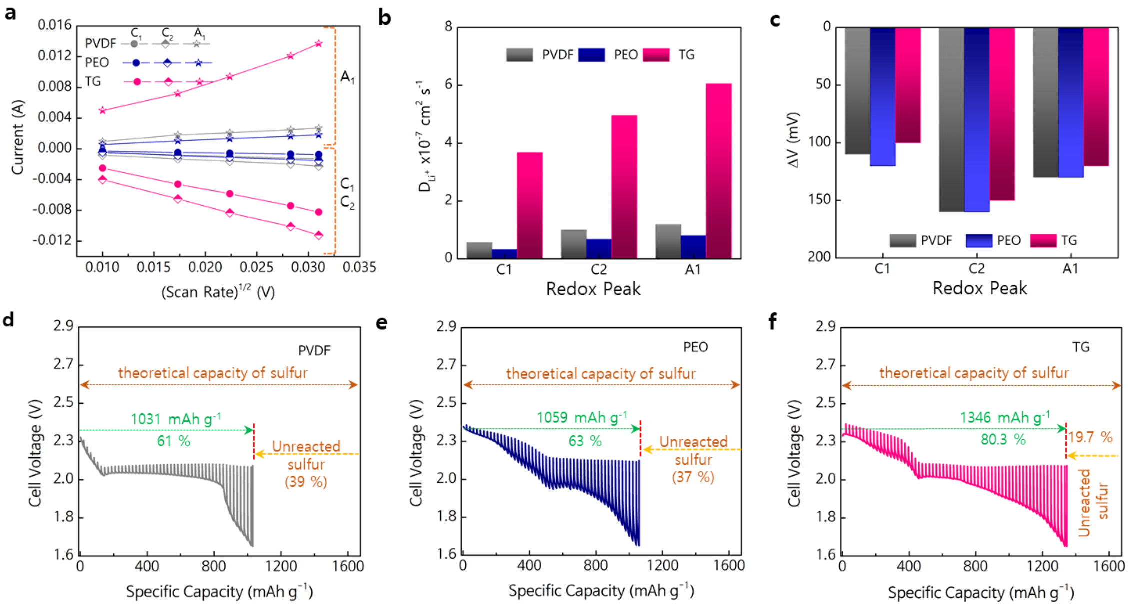

Supplementary Figure 7. Cyclic voltammetry and Galvanostatic intermittent titration technique (GITT) studies. **a**. CV peak current vs. square root of scan rate. **b**. Calculated Li diffusion co-efficient. **c**. Polarized voltage at cathodic (C1, C2) and anodic peaks (A1). **d-f**. GITT discharge profile for the Li-S cells employing S/PVDF, S/PEO, and S/TG electrodes.

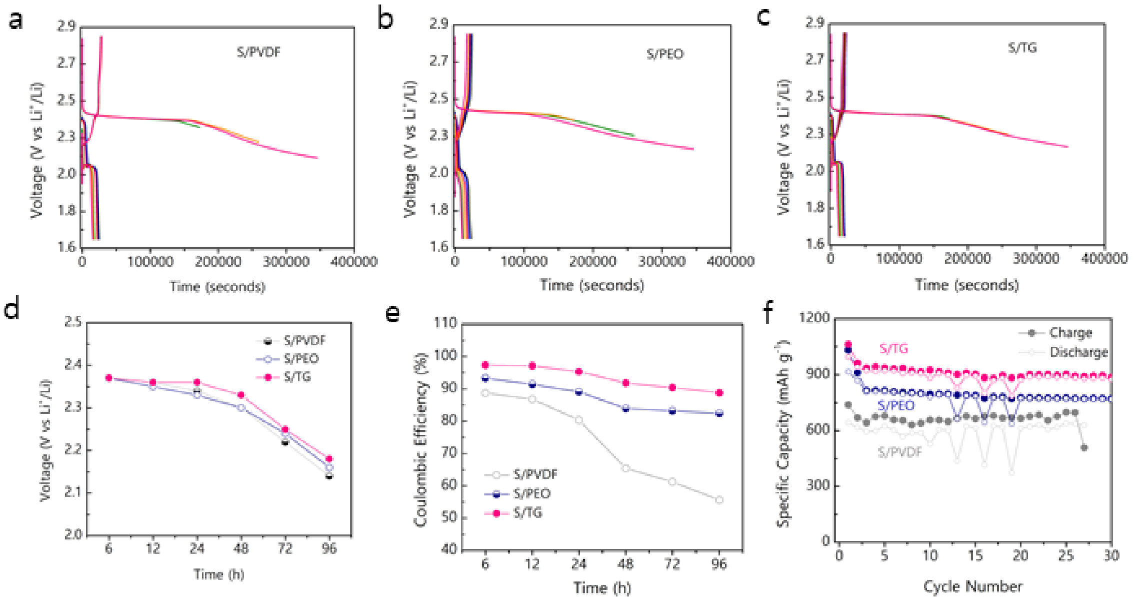

Supplementary Figure 8. Self-discharge behavior evaluated for Li-S cells employing S/PVDF, S/PEO, and S/TG electrodes at a current rate of 0.2 C. **a-c** Galvanostatic discharge-charge profile for S/PVDF, S/PEO, and S/TG cathodes. **d** Plot denoting the change of voltage as a function of resting time. **e** Coulombic efficiency shown for the resting cycle and the recovered cycle measured at various resting times of 6 h -12 h -24 h -48 h -72 h and 96 h. **f** Capacity retention plots of S/PVDF, S/PEO, and S/TG electrodes obtained at a current rate of 0.2 C.

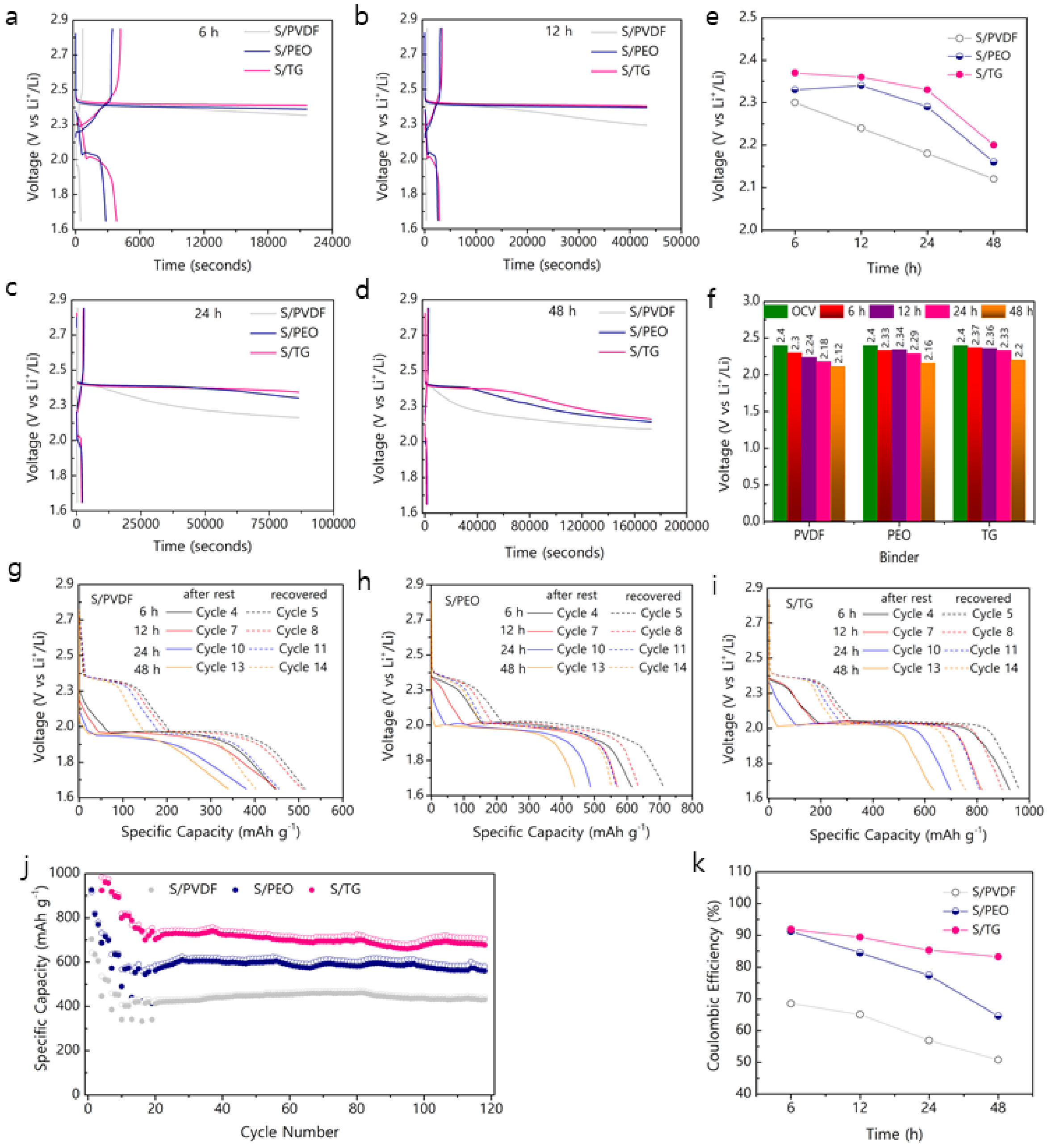

Supplementary Figure 9. Self-discharge behavior evaluated for Li-S cells employing S/PVDF, S/PEO, and S/TG electrodes at a high current rate of 1 C. **a-d** Galvanostatic discharge-charge profile for S/PVDF, S/PEO, and S/TG cathodes at various resting times of 6 h – 12 h – 24 h and 48 h. **e** Plot depicting the change of voltage as a function of resting time. **f** Bar graph portraying the change in voltage as a function of time for Li-S cells employing S/PVDF, S/PEO, and S/TG electrodes. **g-i** Discharge profile shown after resting cycle and the recovery cycle for S/PVDF, S/PEO, and S/TG cathodes at various resting times. **j** Capacity retention plots of S/PVDF, S/PEO, and S/TG electrodes obtained at a high current rate of 1 C. **k** Coulombic efficiency shown for the resting cycle and the recovered cycle measured at various resting times of 6 h -12 h -24 h and 48h.

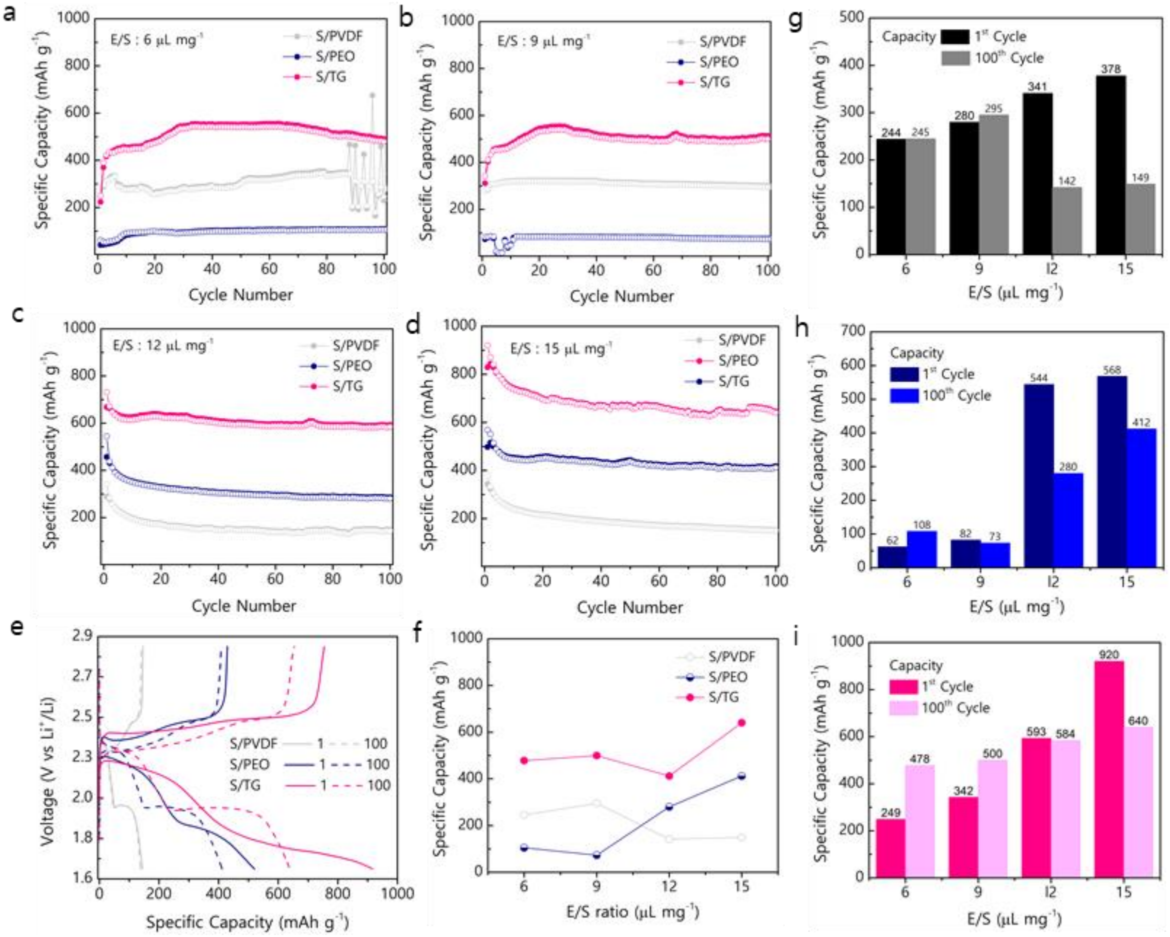

Supplementary Figure 10. Electrochemical performances with specific electrolyte/sulfur (E/S) ratios of 6, 9, 12, and 15 µL mg^{-1} for Li-S cells employing S/PVDF, S/PEO, and S/TG electrodes. Capacity retention and discharge-charge profile plots. **a** S/PVDF. **b** S/PEO. **c** S/TG electrodes obtained at electrode sulfur loading of 2.0-2.1 mg cm^{-2} at a current rate of 1 C. **e** Plot of capacity against E/S ratio. **f-h** Bar graph depicting the 1st and 100th cycle for S/PVDF, S/PEO, and S/TG electrodes at E/S ratio of 6, 9, 12, and 15 µL mg^{-1}.

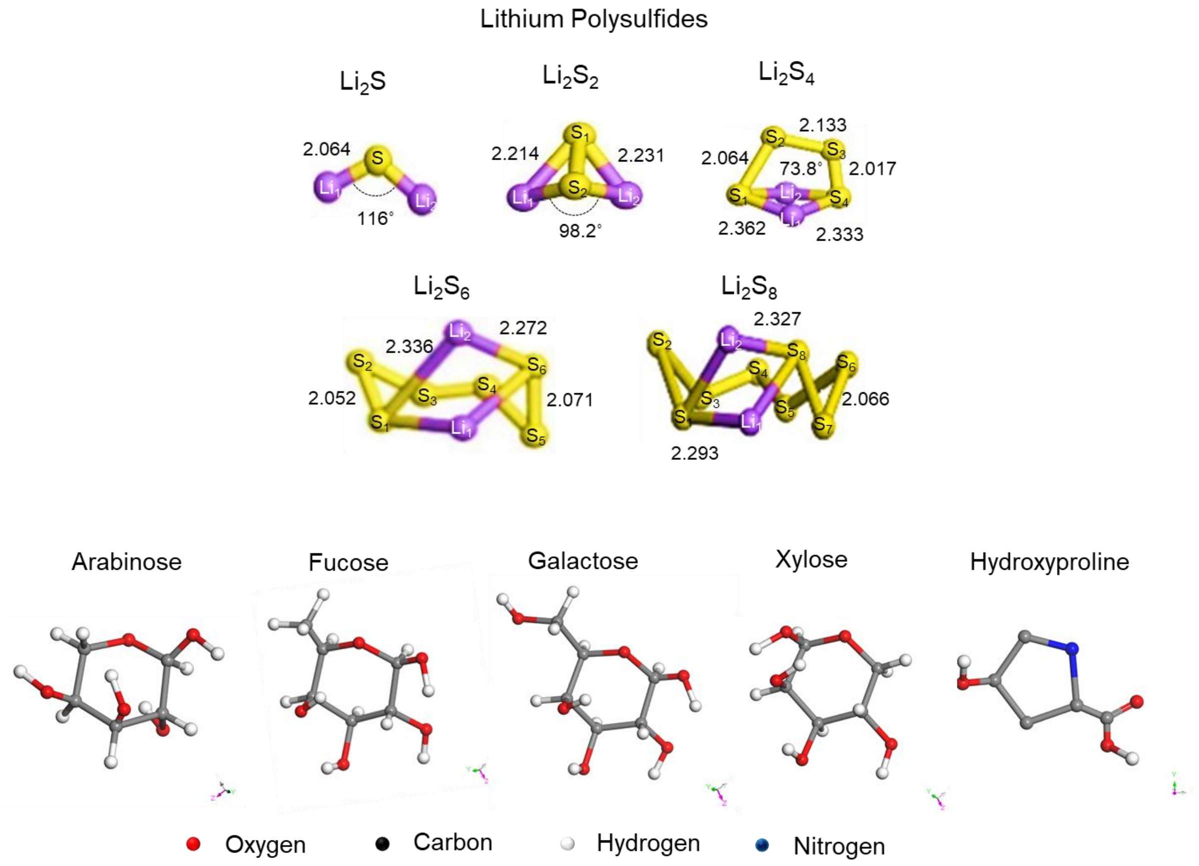

Supplementary Figure 11. Optimized geometric structures of lithium polysulfides, Li_2S, Li_2S_2, Li_2S_4, Li_2S_6, and Li_2S_8 and polysaccharidic units - arabinose, fucose, galactose, xylose, and hydroxyproline used to study the binding energy between polysulfides and saccharadic units.

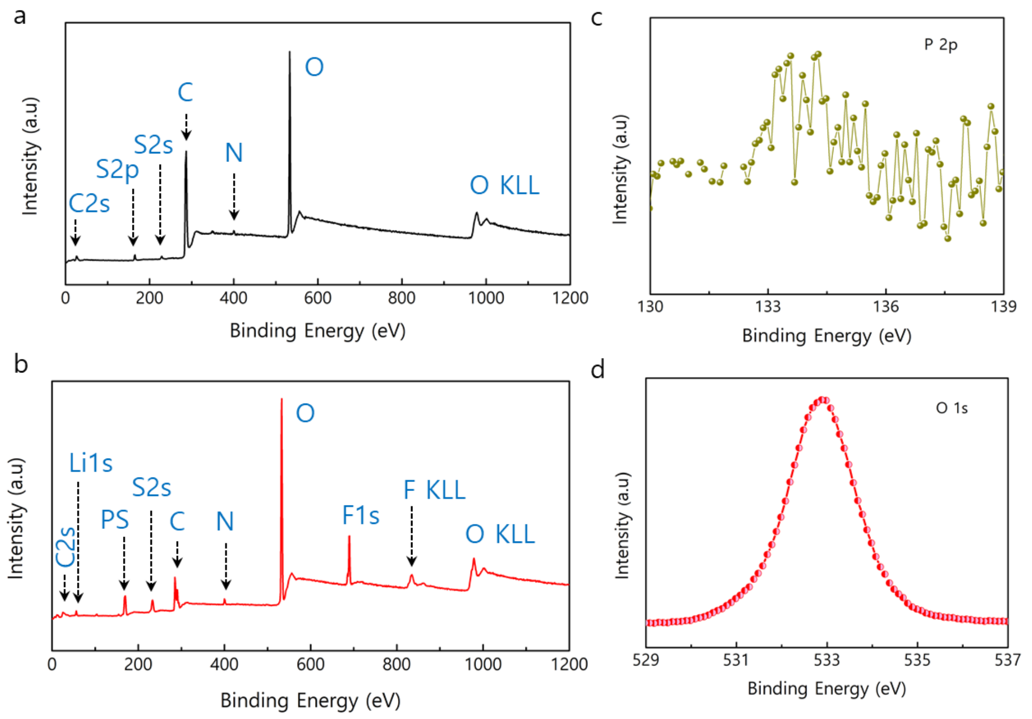

Supplementary Figure 12. XPS studies for S/TG electrodes. **a** Survey scan for the fresh electrode. **b** Survey scan for the cycled electrode. **c-d** P 2p and O1s for postmortem S/TG electrodes.

Supplementary Note 1: Electrode architecture upon cycling

The morphological and mechanical integrity of the sulfur electrodes were studied in surface and cross-section mode observed through field-emission scanning electron microscopy (FE-SEM). The cross-sectional micrographs for all the pristine electrodes using PVDF, PEO, and TG binders confirmed that the average thickness was well controlled at ∼35 µm (Supplementary Figure 13), which was undertaken to have a reasonable comparison. Electrodes subjected to consecutive discharges-charges at a current rate of 4 C up to 1000 cycles were considered for postmortem studies, where all the cycled electrodes showed an increment in the electrode thickness (Supplementary Figure 13a2-c2). It is known that the increase in the electrode thickness upon cycling is associated with the volume expansion of sulfur (ρ=2.03 g cm^{-3}) particles up to 80% and in addition to the deposition of insoluble lithium polysulfides (ρ=1.67 g cm^{-3}). However, the increased thickness of cycled S/TG electrode was about 6 µm; comparatively higher growth is evidenced for S/PVDF and S/PEO electrodes exhibiting about 13 µm and 9 µm, respectively.

The calculated increase in volume expansion from cross-section micrographs was minimal at 16% for S/TG electrodes compared to 37% for S/PVDF and 22% for S/PEO electrodes (Supplementary Fig. 13d). More serious is that the cycled S/PVDF and S/PEO electrodes exposed severe structural disruptions observable as cracks ranging between ∼2.2 to 3.7 µm (Supplementary Fig. 13a3,b3), which occurred in the entire surface. It was revealed that the limited adhesion of the sulfur and conductive particles afforded by PVDF and PEO binders (Supplementary Fig. 13a4,b4) and their poor control over the volume expansion leads to electrode pulverization rooting capacity fade. Apparently, S/TG electrodes maintained an intact surface possessing several voids and micropores that could favor not only better contact with in-depth sulfur particles but also favors facile ion movement and mitigating volume change (Supplementary Fig. 13c3).

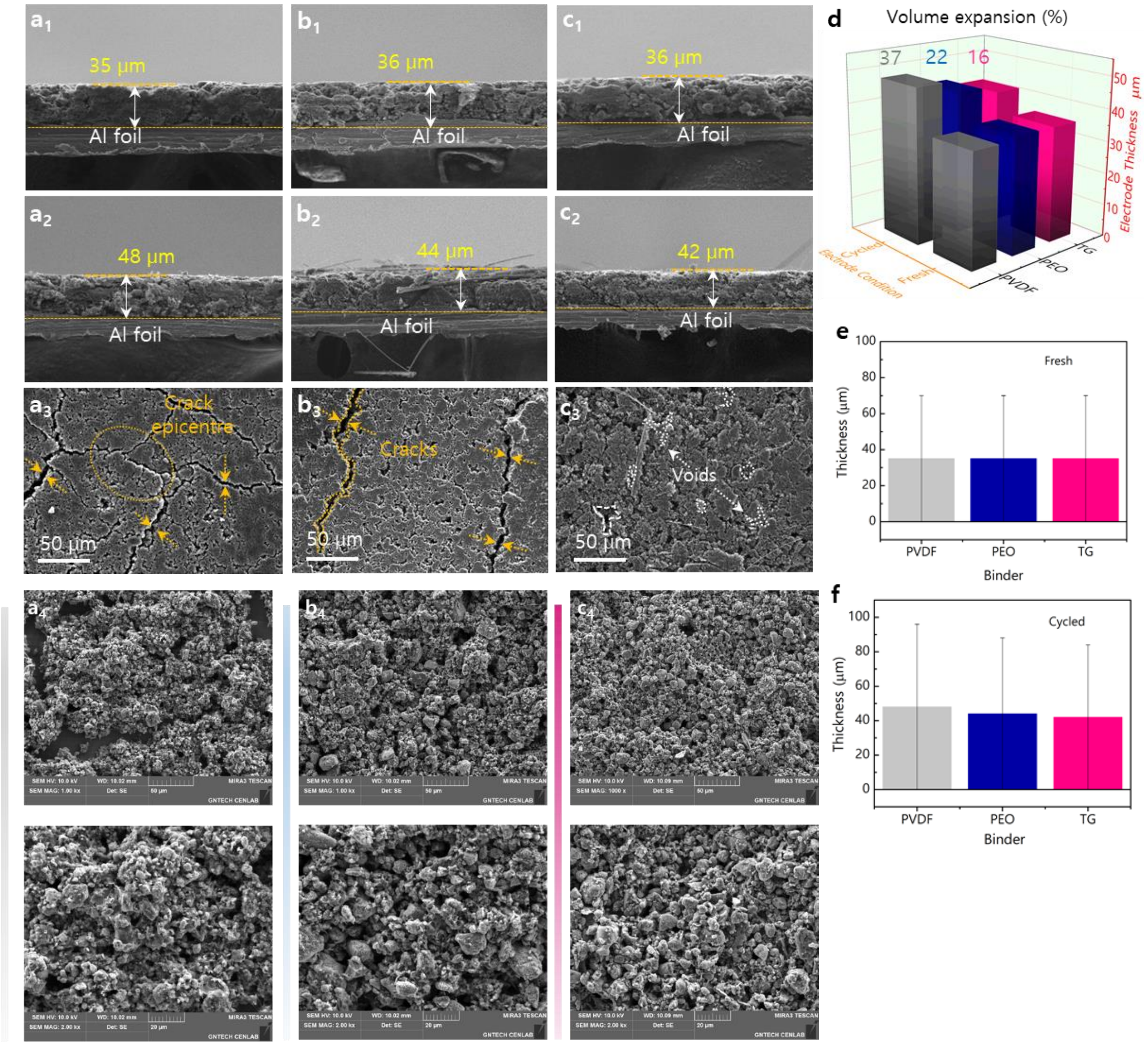

Supplementary Figure 13. Cross-section and surface microscopic analysis through SEM. a_1-c_1 a_2-c_2 Cross-section of fresh and cycled S/PVDF, S/PEO and S/TG electrodes. **a_3-c_3** and **a_4-c_4** Surface microscopic images. **a** S/PVDF. **b** S/PEO. **c** S/TG for cycled and fresh electrodes. **d** 3D view depicting the volume expansion upon cycling. **e-f** Error bar for fresh and cycled S/PVDF, S/PEO, and S/TG electrodes

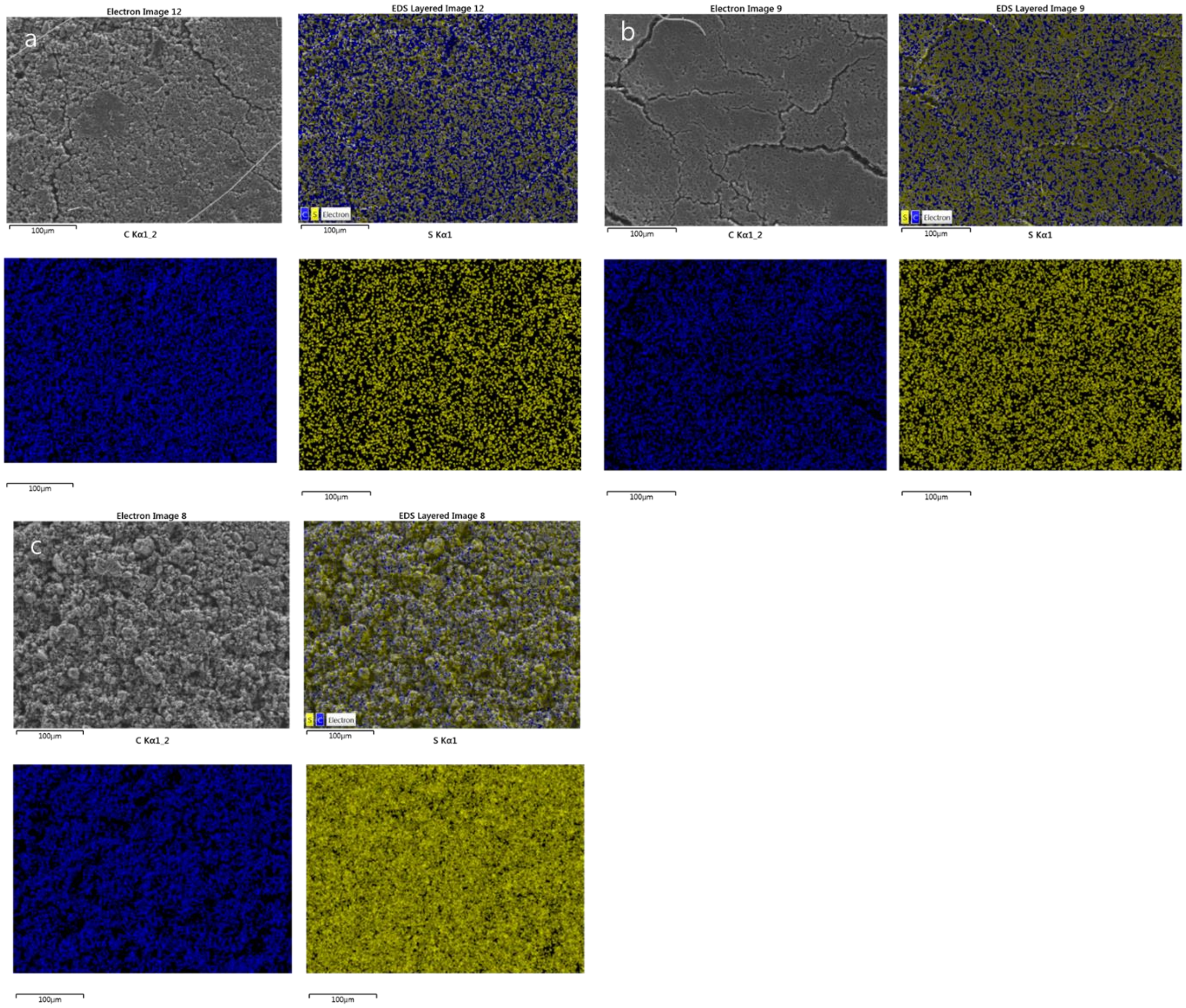

Supplementary Figure 14. EDS mapping for the sulfur cathodes. **a** S/PVDF. **b** S/PEO, and **c** S//TG electrodes recovered after 1000th cycle performed at a current rate of 4 C.

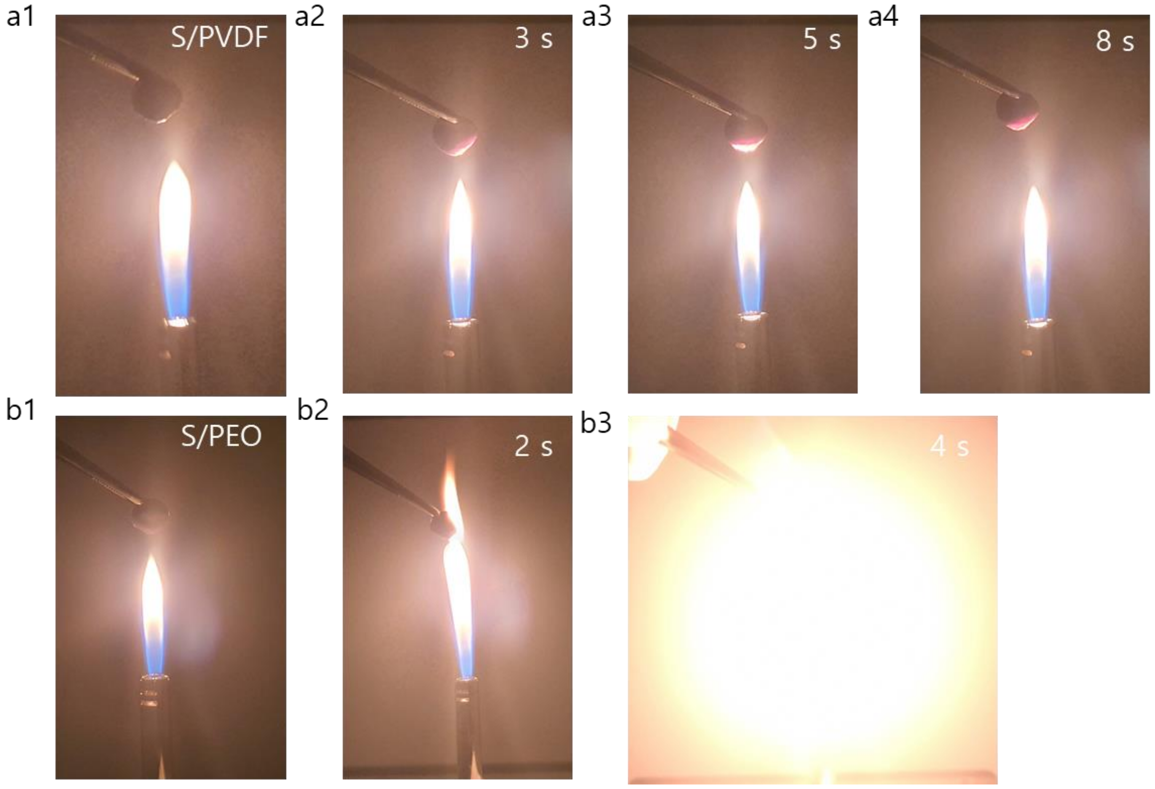

Supplementary Figure 15. Flame-retardant studies of sulfur electrodes. **a** S/PVDF and **b** S/PEO electrodes.

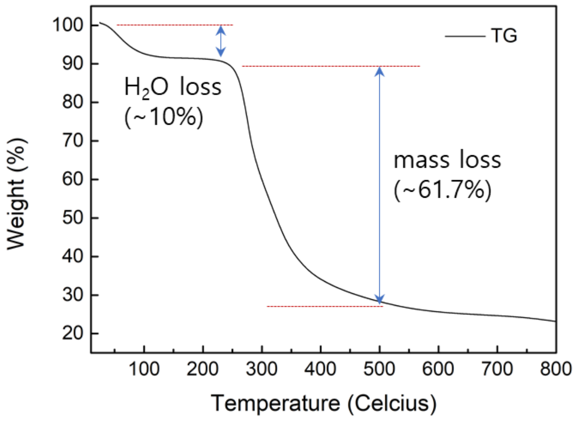

Supplementary Figure 16. Thermal stability of pristine TG binder studied through thermogravimetric analysis (TGA).

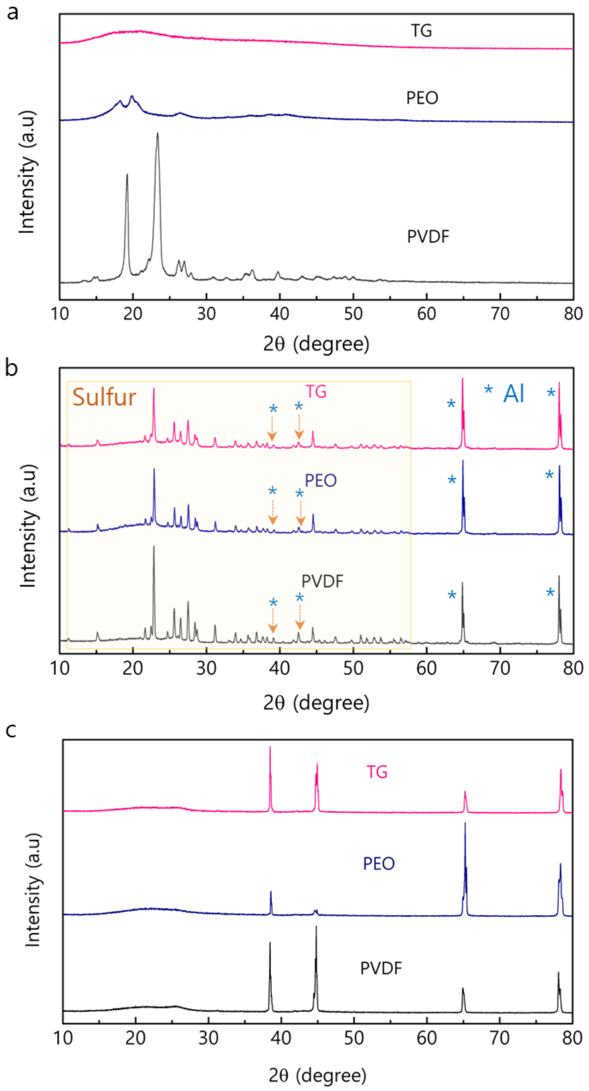

Supplementary Figure 17. X-ray diffraction studies. **a** Pristine binders. **b** Pristine S/PVDF, S/PEO and S/TG electrodes. **c** Respective flame-exposed electrodes.

Supplementary Note 2: Mechanism for flame-retardant nature

X-ray diffraction studies performed for the fresh and after-flame exposed sulfur electrodes are shown in Supplementary Figure 17. The XRD pattern for pristine binders shows crystalline nature for PVDF and PEO, while TG exposed an amorphous state. The electrodes fabricated with binders exhibited peaks corresponding to sulfur and carbon, while binder's peaks are noticeable only for the case of PVDF and PEO electrodes which is in agreement with the pristine binders XRD peaks. Moreover, the electrodes fabricated with TG do not show any crystal features to the TG binder. Upon flame-exposed, all the sulfur cathodes exposed peaks respective of aluminium current collector, however the PVDF and PEO-based electrodes lost their crystallinity upon ignition causing serious damage to the electrodes as they succumb to more-flammable characteristics. In the case of TG, the amorphous gum which possesses abundant -OH functional groups upon contact with the flame (temperature ~180.3 ℃) tend to lose water molecules forming a stable protective layer which would have acted as a protection and thereby suppress the sulfur active material from reacting vigorously to the flame (Figure 7g and Supplementary Figure 15) which is why the TG-based electrodes showed good electrode architecture (Figure 7g). From the flame-retardant studies, it is known that the amorphous nature of tragacanth in conjunction with other electrode constituents remained passive to the flame, while other PVDF and PEO electrodes reacted to flame. Thus, the property of TG employed as a binder in sulfur cathode would have largely benefitted from the characteristics. In addition, the surface digital photographs of fresh and flame-exposed electrodes (Figure 7g) revealed an intact electrode characteristic for TG binder employed sulfur cathode among the PVDF and PEO sulfur cathodes. This intact feature is corroborated with an amorphous nature of TG (before and after flame) noticeable from the XRD, which is related to the formation of a passive layer upon interaction with flame.

Supplementary Table 1. Comparison of sulfur mass loading for variously reported binders in Li-S batteries (C-rate based on sulfur's theoretical capacity 1.675 A g^{-1}).

Cell parameter				Cell performance						
**Binder (B)**	**Electrode ratio (%) S/C/B**	**Sulfur load (mg cm^{-2})**	**E/S ratio (μL mg^{-1})**	**Rate (C)**	**Areal Capacity ($mAh\ cm^{-2}$)**		**Cycle no.**	**Capacity retention (%)**	**Current collector**	**Ref.**
					Initial	Final				
PVDF	7:2:1	2.0	-	0.4	1.8	1.62	50	90	Ni foam	6
Gelatin	6.3:3:0.7	2.0	-	0.4	2.3	0.8	50	34	Al foil	7
PAMAC	8:1:1	3.0	-	0.5	3.2	2.0	105	62.5	C-coated Al foil	8
DCP	6.4:2.6:0.1	9.8	5	0.5	9.2	6.5	50	70.6	Ni foam	9
CDp-N+	7:2:1	5.5	7	0.3	6.6	4.4	45	66.6	Al foil	10
MPEII	5:4:1	6.5	-	0.05	7.52	5	70	66.4	Al foil	11
PAMAM	8:1:1	4.0	40	0.05	4.56	2.8	100	61.4	-	12
S/GG-XG	8:1:1	6.5	21	0.8	4.2	~4.7	90	101	-	13
Carrageenan	-	5-10	-	0.01	~9	~5.8	100	64.4	-	14
CMC-CA	8.5:4.5:5.5	10	4	1.0	~11.2	~5.7	50	50.8	Carbon paper	15
PEI	6:3:1	8.6	16	0.05	9.7	6.4	50	65.9	Al foil	16
**Tragacanth gum**	**8:1.5:0.5**	**8.1**<br>**4.7**	**12-15**	**0.1**<br>**0.1**	**10.8**<br>**9.7**	**10.02**<br>**6.3**	**100**	**92.7**<br>**64.9**	**Al foil**<br>**Al foil**	**Our Work**

Supplementary Table 2. Comparison of Li-S battery performance and its stability using various bio-based and conventional binders (C-rate based on sulfur's theoretical capacity 1.675 A g^{-1}).

		Cell parameter			Cell performance					
**Type of polymer binder**	**Binder (B)**	**Electrode ratio (%) S/C/B**	**Sulfur load (mg cm^{-2})**	**E/S ratio (µL mg^{-1})**	**Rate (C)**	**Specific capacity (mAh g^{-1})**		**Cycle no.**	**Capacity decay per cycle**	**Ref.**
						Initial	Final			
Organic	PVDF	7:2:1	2.4	50	0.5	788	322	100	0.49	17
	PAA	6:3:1	1.5	-	0.2	390	298	100	0.09	18
	PAA/ PEDOT:PSS	7:2:1	0.8	-	0.5	1121	830	80	0.36	19
	PVA	6:3:1	3.5	9	0.5	936	563	75	0.49	20
Bio-based	Gum arabic	8:2	1.5	100	0.2	1157	841	500	0.054	21
	Guar/ Xanthum	8:1:1	6.5	21	0.5	1000	724	150	0.18	10
	Na-alginate	5:4:1	1.5	-	0.5	776	508	50	0.53	22
	Gelatin	6.3:3:0.7	2.0	-	0.5	950	680	100	0.28	8
	Zein/CNF	6.38:2.41: 0.21	1.8	-	0.6	890	590	500	0.06	23
	SBR: CMC	5.4:3.6:1	1	-	0.2	867	612	150	0.55	24
	Chitosan	6.3:3:0.7	1-1.5	-	0.2	1145	780	100	0.36	25
	Gelatin/ Chitosan	6.3:3:0.7	1-1.5	-	0.5	857	675	200	0.09	27
	Soy protein-PAA	6.75:2.25: 0.1	1.5	10	0.3	790	595	200	0.12	26
	LA-132	7:2:1	2	80	0.5	901	470	100	0.43	27
	LA-133	5.4:3.6:1	1	-	0.2	1176	709	150	0.31	22
	Catechol-Chitosan sulfate	8.5:0.5:1	1.3-1.5	15	0.2	785	605	400	0.045	28
	Laponite	8:1:1	1	-	0.5	810	595	500	0.05	29
	Polyurethane -Phytic acid	-	0.8-1.5	25	0.5	1051	632	500	0.08	30
	Cyclodextrin -S composite	7:2:1	2.5	7	0.5	692	636	50	0.02	AFM
	**Tragacanth gum**	**8:1.5:0.5**	**1.1-1.4**	**15-17**	**0.2<br>1**	**1239<br>1236**	**976<br>865**	**200<br>300**	**0.13<br>0.12**	**Our work**

Supplementary Table 3. Wettability test performed for the S/PVDF, S/PEO, and S/TG electrodes.

Electrodes \ Time	Mass at 0 h	Mass after 24 h	[%] increase
S/PVDF	5.1 mg	11.1 mg	117.6
S/PEO	6.9 mg	16.9 mg	144.92
S/TG	6.7 mg	20.9 mg	211.94

The wettability measurement taken after 24 h of steeping in electrolyte included traces of electrolyte solution stuck to the electrode surfaces.

Supplementary Table 4. Comparison of binding energy values obtained through DFT analyses for several reported metals, metalloid, semi-metal, and polymeric binders with the TG binder in Li-S batteries.

Class of Material	Bonding type	Binding energy (eV)	Electrode material (Sulfur+Host or Additive)	Capacity after nth cycle	Ref.
Non-metal	S–C	-1.64–2.84	C/S host	860 (500)	31
	S–O	-0.84–1.95	O-CNTs/S host	800 (600)	32
	Li_2S_x–N	-0.25–4.08	N-doped C/Graphene<br>g-C_3N_4/S composite	752 (300)<br>620 (500)	33<br>34
	Li_2S_x–P	-0.94–1.39	P-doped CNTs,<br>N, P-doped graphene	917 (100)<br>638 (500)	35<br>36
Metalloid	Li_2S_x–B	-0.9–5.15	V-doped C coated CNTs/S<br>N, B-doped C/S	562 (500)<br>556 (500)	37<br>38
Metal	S-Ti	-2.33	S in porous C/TiO_2<br>Ti_4O_7/S	800 (200)<br>800 (250)	39<br>40
	Li_2S_x–V	-1.9	V_2O_3/C<br>V_2O_5/C	921 (100)<br>900 (100)	41<br>42
	S–Mn	-1.84–2.59	MnO_2/S<br>S coated MnO_2 sheets	802 (300)<br>1030 (200)	43<br>44
	S–Fe	-0.84–1.85	FeS_2 in S/C<br>Fe_2O_3 in S	700 (200)<br>575 (100)	45<br>46
	S–Co	-3.79	CoS_2 in Graphene/S<br>Co_3O_4-S	750 (250)<br>656 (200)	47<br>48
	S–Ni	-0.72–2.59	S/NiS@C spheres<br>nanocrystal S@NiS_2	718 (200)<br>954 (1200)	49<br>50
	S–Cu	-1.56	S/Cu<br>S/C aerogel with CuS	1300 (80)<br>1073 (100)	51<br>52
Polymer	Li–F	-0.4	PVDF	~510 (100)	53
	O–Li/N–Li	-1.81/	PPA	~740 (400)	54
	Li–O	-1.14	PVP	760 (100)	52
	Li–O	-0.52	PVA	515 (250)	21
	Li–O	-	CMC	~390 (350)	57
	Li–N	-1.24	PEI	~930 (100)	55
	Li–N	-0.59	PANI	628 (200)	56,57
	O–Li/S–Li	-3.59/-2.47	DSM	686 (350)	58
	Li–O	-2.17	APP	640 (400)	59
	**O–Li/N–Li**	**-1.17/-1.21**	**Tragacanth**	**976 (200)**	**Our work**

Supplementary Table 5. Comparison of flexible battery performance

**Anode**	**Cathode**	**Energy density ($Wh\ L^{-1}$)**	**Cycle number**	**Ref.**
Li foil/CNT	S/CNT/PVDF	115	3	60
Li foil	S/Graphene	39	20	61
Li foil	S/Graphene nanotube	-	60	62
Li foil	S/CB/Gr-PP/PVDF	50	30	63
Li foil	S/Graphene/Cotton	-	50	64
Li foil	Li_2S_6/Carbon cloth	-	10	[65]
Li foil	S/Graphene/PEDOT	120	80	66
Li foil	S/MWCNT/MnO_2@PP	-	20	67
**Li foil**	**S/MWCNT/TG**	**230**	**50**	**Our work**

Supplementary Table 6. Resistance values derived from the respective Nyquist plots for S/PVDF, S/PEO and S/TG electrodes.

Electrode	R_s (ohm)	R_{ct} (ohm)
S/PVDF	10.06	249.34
S/PEO	6.4	211.69
S/TG	7.9	125.19

Supplementary Table 7. Tap density measurements for S/PVDF, S/PEO, and S/TG cathodes with electrode constituent ratio 8: 1.5: 0.5 (S/C: MWCNT: Binder).

Test	S/C: MWCNT: PVDF		S/C: MWCNT: PEO		S/C: MWCNT: TG	
	Density (g cm^{-3})	Error	Density (g cm^{-3})	Error	Density (g cm^{-3})	Error
A	1.0862	0.00008	1.0643	0.00064	1.0863	0.0014
B	1.0853	0.00082	1.0623	0.00264	1.0848	0.0001
C	1.0883	0.00218	1.0668	0.00186	1.0822	0.0041
D	1.0861	0.00002	1.0647	0.00024	1.0857	0.0008
E	1.0847	0.00142	1.0666	0.00166	1.0855	0.0006
Average	1.08612	0.000904	1.06494	0.001408	1.0849	0.007

Supplementary Table 8. SEM thickness estimates for fresh and cycled S/PVDF, S/PEO, and S/TG electrodes.

Fresh electrodes

Fresh Test	S/C: MWCNT: PVDF		S/C: MWCNT: PEO		S/C: MWCNT: TG	
	thickness (μm)	Error	thickness (μm)	Error	thickness (μm)	Error
A	34.95	0.056	36.00	0.016	36.00	0.05
B	35.00	0.006	36.10	0.084	36.10	0.05
C	35.05	0.044	36.10	0.084	36.00	0.05
D	35.01	0.004	36.00	0.016	36.10	0.05
E	35.02	0.014	36.90	0.116	36.00	0.05
F	35.01	0.004	36.00	0.016	36.10	0.05
Average	35.006	0.0213	36.016	0.0553	36.05	0.05

Cycled electrodes

Cycled Test	S/C: MWCNT: PVDF		S/C: MWCNT: PEO		S/C: MWCNT: TG	
	thickness (μm)	Error	thickness (μm)	Error	thickness (μm)	Error
A	48.00	0.016	44.00	0.058	42.00	0.016
B	48.05	0.034	44.00	0.058	42.10	0.084
C	48.00	0.016	44.05	0.008	42.00	0.016
D	48.00	0.016	44.10	0.042	42.00	0.016
E	47.95	0.066	44.10	0.042	42.00	0.016
F	48.10	0.084	44.10	0.042	42.00	0.016
Average	48.016	0.0386	44.058	0.0416	42.016	0.0273

Parameter for the flexible Li-S battery

Weight		Mass loading		Size	
Anode	0.1086 g	Anode (Li)	3.0 mg cm^{-2}	Anode	4 × 4
Cathode	0.1428 g	Cathode (S)	3.84 mg cm^{-2}	Cathode	4 × 4
Separator	0.0293 g	Electrolyte/Sulfur ratio	8 µL mg^{-1}	Separator	4.3 × 4.3
Overall cell		Cell thickness	360 µm		

Total energy

Energy (E) = Capacity (C) × Voltage (V)

E = 59.31 mAh × 2.24 V = 132.85 mWh

Areal energy (E_a)

E_a = Energy (E) / Area (A)

E_a = 132.85 / 16 cm^2 = 8.303 mWh cm^{-2}

Volumetric energy density (Ev)

E_v = Areal capacity (E_a) / Cell thickness

E_v = 8.303 / 360 = 230 Wh L^{-1}

Gravimetric energy density (E_G)

E_v = Areal capacity (E_a) / Cell mass

E_v = 8.303 / 341 = 243 Wh kg^{-1}

Supplementary References

1 Mikhaylik, Y.V. & Akridge, J.R. Polysulfide shuttle study in the Li/S battery system. *J. Electrochem. Soc.* **151**, A1969-A1976 (2004).

2 Huang, J.Q. Zhang, Q. Zhang, S.M. et al. Aligned sulfur-coated carbon nanotubes with a polyethylene glycol barrier at one end for use as a high efficiency sulfur cathode. *Carbon* **58**, 99-106 (2013).

3 Huang, J.Q. et al. Permselective graphene oxide membrane for highly stable and anti-self-discharge lithium-sulfur batteries. *ACS Nano* **9**, 3002-3011 (2015).

4 Wang, Y. et al. Inhibiting polysulfides shuttling by dual-functional nanowires/nanotubes modified layers for highly stable lithium-sulfur batteries. *New J. Chem.* **43**, 14708-14713 (2019).

5 Nejatian, M. Abbasi, S. Azarikia, F. Gum Tragacanth: Structure, characteristics and applications in foods. *Int. J. Biol. Macromol.* **160**, 846–860 (2020).

6 Chung, S. Manthiram, A. Lithium–sulfur batteries with superior cycle stability by employing porous current collectors. *Electrochim. Acta* **107**, 569-576 (2013).

7 Sun, J. et al. Application of gelatin as a binder for the sulfur cathode in lithium-sulfur batteries. *Electrochim Acta* **53**, 7084-7088 (2008).

8 Zhang, S. S. Binder Based on polyelectrolyte for high capacity density lithium/sulfur battery. *J. Electrochem. Soc.* **159**, A1226 (2012).

9 Liu, X. et al. Greatly improved conductivity of double-chain polymer network binder for high sulfur loading lithium–sulfur batteries with a low electrolyte/sulfur ratio. *Small* **14**, 1801536 **(**2018**).**

10 Zeng, F. et al Multidimensional polycation β-cyclodextrin polymer as an effective aqueous binder for high sulfur loading cathode in lithium-sulfur batteries. *ACS Appl. Mater. Inter.* **7**, 26257 (2015).

11 Wang, H. et al. Cationic polymer binder inhibit shuttle effects through electrostatic confinement in lithium sulfur batteries. *J. Mater. Chem. A* **6**, 6959-6966 (2018).

12 Hong, X. et al. On the dispersion of lithium-sulfur battery cathode materials effected by electrostatic and stereo-chemical factors of binders. *J. Power Sources* **324**, 455 (2016).

13 Liu, J. et al. Exploiting a robust biopolymer network binder for an ultrahigh-areal-capacity Li–S battery. *Energy Environ. Sci.* **10**, 750–755 (2017).

14 Ling, M. et al. Nucleophilic substitution between polysulfides and binders unexpectedly stabilizing lithium sulfur battery. *Nano Energy* **38**, 82–90 (2017).

15 Pang, Q. et al. A comprehensive approach toward stable lithium–sulfur batteries with high volumetric energy density. *Adv. Energy Mater.* **7**, 1601630 (2017).

16 Zhang, L. et al. Effective electrostatic confinement of polysulfides in lithium/sulfur batteries by a functional binder. *Nano Energy* **40**, 559–565 (2017).

17 Eom, J.Y. Kim, S.I. Ri, V. Kim, C. The effect of polymeric binders in the sulfur cathode on the cycling performance for lithium–sulfur batteries. *Chem. Commun.* **55**, 14609-14612 (2019).

18 Zhang, Z. et al. Water-soluble polyacrylic acid as a binder for sulfur cathode in lithium-sulfur battery. *ECS Electrochemistry Lett* **1**, A34 (2012).

19 Pan, J. et al. PAA/PEDOT:PSS as a multifunctional, water-soluble binder to improve the capacity and stability of lithium–sulfur batteries. *RSC Adv.* **6**, 40650-40655 (2016).

20 Liao, J. Liu, Z. Wang, J. Ye, Z. Cost-effective water-soluble poly(vinyl alcohol) as a functional binder for high-sulfur-loading cathodes in lithium–sulfur batteries. *ACS Omega* **5**(14), 8272-8282 (2020).
21 Li, G. et al. Acacia senegal–inspired bifunctional binder for longevity of lithium sulfur batteries. *Adv. Energy Mater.* **4**, 1500878 (2015).
22 Bao, W. Zhang, Z. Gan, Y. Wang, X, Lai. J. Enhanced cyclability of sulfur cathodes in lithium-sulfur batteries with Na-alginate as a binder. *J Energy Chemistry* **22**, 790-794 (2013).
23 Chen, M. et al. Let it catch: A short-branched protein for efficiently capturing polysulfides in lithium–sulfur batteries. *Adv Energy Mater* **10**, 1903642 (2020).
24 Wang, W. et al. Comparative study of water-based LA133 and CMC/SBR binders for sulfur cathode in advanced lithium–sulfur batteries. *J. Phys. Chem. C* **123**, 250–257 (2019).
25 Chen, Y. et al. Chitosan as a functional additive for high-performance lithium–sulfur batteries. *J. Mater. Chem. A* **3**, 15235-15240 (2015).
26 Fu, X. Scudiero, L. Zhong. W.H. A robust and ion-conductive protein-based binder enabling strong polysulfide anchoring for high-energy lithium–sulfur batteries. *J. Mater. Chem. A* **7**, 1835-1848 (2019).
27 Pan, J. Xu, G. Ding, B. Han, J. Dou, H. Zhang, X. Enhanced electrochemical performance of sulfur cathodes with a water-soluble binder. *RSC. Adv.* **5**, 13709-13714 (2015).
28 Yi, H. et al. Aqueous-processable polymer binder with strong mechanical and polysulfide-trapping properties for high performance of lithium–sulfur batteries. *J. Mater. Chem. A* **6**, 18660-18668 (2018).
29 Guo, R. Wang, J. Zhang, S. Han, W.Q. Multifunctional cross-linked polymer-Laponite nanocomposite binder for lithium-sulfur batteries. *Chem. Eng. J* **388**, 124316 (2020).
30 Man, L. et al. In situ-cross-linked supramolecular eco-binders for improved capacity and stability of lithium–sulfur batteries. ACS Appl. Energy Mater. **4**, 3803-3811 (2021).
31 Zheng, S. et al. High performance C/S composite cathodes with conventional carbonate-based electrolytes in Li-S battery. *Sci. Rep.* **4**, 4842 (2014).
32 Song, J. et al. Nitrogen-doped mesoporous carbon promoted chemical adsorption of sulfur and fabrication of high-areal-capacity sulfur cathode with exceptional cycling stability for lithium-sulfur batteries. *Adv. Funct. Mater.* **24**, 1243-1250 (2014).
33 Qiu, Y. et al. High-rate, ultralong cycle-life lithium/sulfur batteries enabled by nitrogen-doped graphene. *Nano Lett.* **14**, 4821-4827 (2014).
34 Pang, Q, Nazar, L.F. Long-life and high-areal-capacity Li-S batteries enabled by a light-weight polar host with intrinsic polysulfide adsorption. *ACS Nano* **10**, 4111-4118 (2016).
35 Guo, M.Q. et al. Hydrothermal synthesis of porous phosphorus-doped carbon nanotubes and their use in the oxygen reduction reaction and lithium-sulfur batteries. *New Carbon Mater.* **31**, 352-362 (2016).
36 Gu, X. et al. A porous nitrogen and phosphorous dual doped graphene blocking layer for high performance Li–S batteries. *J. Mater. Chem. A* **3**, 16670-16678 (2015).
37 Wu, F. et al. Boron-doped microporous nano carbon as cathode material for high-performance Li-S batteries. *Nano Res.* **10**, 426-436 (2017).
38 Yuan, S. et al. Graphene-supported nitrogen and boron rich carbon layer for improved performance of lithium–sulfur batteries due to enhanced chemisorption of lithium polysulfides. *Adv. Energy Mater.* **6**, 1501733 (2016).

39 Evers, S. Yim, T. Nazar, L.F. Understanding the nature of absorption/adsorption in nanoporous polysulfide sorbents for the Li–S battery. *J. Phys. Chem. C* **116**, 19653-19658 (2012).

40 Pang, Q. Kundu, D. Cuisinier, M. Nazar. L.F. Surface-enhanced redox chemistry of polysulphides on a metallic and polar host for lithium-sulphur batteries. *Nat. Commun.* **5**, 4759 (2014).

41 Tang, R. Li, X. Ding, Z. Zhang, L. An ultrafine V_2O_3 modified hierarchical porous carbon microsphere as a high performance cathode matrix for lithium–sulfur batteries. *RSC Adv.* **6**, 65162-65170 (2016).

42 Kim, M.S. et al. The effect of V_2O_5/C additive on the suppression of polysulfide dissolution in Li-sulfur batteries. *J. Electrochem. Soc.* **33**, 142-148 (2014).

43 Ni, L. et al. Core–shell structure and interaction mechanism of γ-MnO_2 coated sulfur for improved lithium-sulfur batteries. *Small* **13**, 1603466 (2017).

44 Liang, X. et al. A highly efficient polysulfide mediator for lithium–sulfur batteries. *Nat. Commun.* **6**, 5682 (2015).

45 Zhang, S.S. Tran, D.T. Pyrite FeS_2 as an efficient adsorbent of lithium polysulphide for improved lithium–sulphur batteries. *J. Mater. Chem. A* **4**, 4371-4374 (2016).

46 Zhao, C. et al. Pyrite FeS_2 as an efficient adsorbent composite cathode for lithium–sulfur batteries. *Mater. Lett.* **137**, 52-55 (2014).

47 Yuan, Z. et al. Powering lithium–sulfur battery performance by propelling polysulfide redox at sulfiphilic hosts. *Nano Lett.* **16**, 519-527 (2016).

48 Wang, H. et al. Ultrathin cobaltosic oxide nanosheets as an effective sulfur encapsulation matrix with strong affinity toward polysulfides. *ACS Appl. Mater. Interfaces* **9**, 4320-4325 (2017).

49 Ye. C, et al. A 3D hybrid of chemically coupled nickel sulfide and hollow carbon spheres for high performance lithium–sulfur batteries. *Adv. Funct. Mater.* **27**, 1702524 (2017).

50 Liu. Z, et al. High performance Li–S battery based on amorphous NiS_2 as the host material for the S cathode. *J. Mater. Chem. A* **4**, 13395-13399 (2016).

51 Jia. L, et al. Polysulfides capture-copper additive for long cycle life lithium sulfur batteries. *ACS Appl. Mater. Interfaces* **8**, 30248-30255 (2016).

52 Li. X, et al. CuS quantum dot modified carbon aerogel as an immobilizer for lithium polysulfides for high-performance lithium–sulfur batteries. *RSC Adv.* **6**, 71319-71327 (2016).

53 She, Z.W. et al. Stable cycling of lithium sulfide cathodes through strong affinity with a bifunctional binder. *Chem. Sci.* **4**, 3673-3677 (2013).

54 Chen. W, et al. A New Hydrophilic Binder Enabling Strongly Anchoring Polysulfides for High-Performance Sulfur Electrodes in Lithium-Sulfur Battery. *Adv. Energy Mater.* 8, 1702889 (2018).

55 Ma. L, et al., Enhanced Li–S batteries using amine-functionalized carbon nanotubes in the cathode. *ACS Nano* **10**, 1050-1059 (2015).

56 Zhou. W, et al. Yolk–shell structure of polyaniline-coated sulfur for lithium–sulfur batteries. *J. Am. Chem. Soc.* **135**, 16736-16743 (2013).

57 W, Li. et al. Understanding the role of different conductive polymers in improving the nanostructured sulfur cathode performance. *Nano Lett.* **13**, 5534-5540 (2013).

58 Jin. B, et al. Bioinspired binders actively controlling ion migration and accommodating volume change in high sulfur loading lithium–sulfur batteries. *Adv. Energy Mater.* **9**, 1902938 (2019).

59 Zhou. G, et al. An aqueous inorganic polymer binder for high performance lithium–sulfur batteries with flame-retardant properties. *ACS Cent. Sci.* **4**, 260-267 (2018).
60 L. Li, Z.P. Wu, H. Sun, D. Chen, J. Gao, S. Suresh. P. Chow, C.V. Singh, N. Koratkar. A foldable lithium-sulfur battery. *ACS Nano* **9**, 11342-11350 (2015).
61 Wu, C. Fu, L. Maier, J. Yu, Y. Free-standing graphene based porous carbon films with three-dimensional hierarchical architecture for advanced flexible Li-S batteries. *J. Mater. Chem. A* **3**, 9438-9445 (2015).
62 Chen, K. et al. Sulfur nanoparticles encapsulated in reduced graphene oxide nanotubes for flexible lithium-sulfur batteries. *Nano Res.* **11**, 1344-1357 (2018).
63 Zhou, G. et al., A flexible sulfur-graphene-polypropylene separator integrated electrode for advanced Li-S batteries. *Adv. Mater.* **27**, 641-647 (2015).
64 Gao, Z. Zhang, Y. Song, N. Li. X. Towards flexible lithium-sulfur battery from natural cotton textile. *Electrochim. Acta* **246**, 507-516 (2017).
65 Song, J.Y. et al. A polysulfide-infiltrated carbon cloth cathode for high-performance flexible lithium-sulfur batteries. *Nanomaterials* **8**, 90 (2018).
66 Xiao, P. Bu, F. Yang, G. Zhang Y. Xu, Y. Integration of graphene, nano sulfur, and conducting polymer into compact, flexible lithium-sulfur battery cathodes with ultrahigh volumetric capacity and superior stability for foldable devices. *Adv. Mater.* **29**, 1703324 (2017).
67 Yao, M. et al. A flexible all-in-in one lithium-sulfur battery. *ACS Nano* **12**, 12503-12511 (2018).
